# Management of suspected common bile duct stones on cholangiogram during same-stay cholecystectomy for acute gallstone-related disease

**DOI:** 10.1186/s12893-017-0232-z

**Published:** 2017-04-17

**Authors:** Sandra de Sousa, Olivier Tobler, Pouya Iranmanesh, Jean-Louis Frossard, Philippe Morel, Christian Toso

**Affiliations:** 1 Division of Abdominal Surgery, Geneva University Hospitals, Faculty of Medicine, Rue Gabrielle-Perret-Gentil 4, 1205, Geneva, Switzerland; 2 Division of Gastro-intestinal Disease and Hepatology, Geneva University Hospitals, Faculty of Medicine, Geneva, Switzerland; 3 Hepato-pancreatico-biliary Center, Geneva University Hospitals, Faculty of Medicine, Geneva, Switzerland

**Keywords:** Suspected common bile duct stone, Same-stay cholecystectomy for acute gallstone-related disease, Filling defect on intra-operative cholangiogram, Trans-cystic drain

## Abstract

**Background:**

Recent data have suggested that upfront cholecystectomy should be performed even in the presence of moderately abnormal liver function tests (LFTs). As a consequence, more common bile duct (CBD) stones are discovered on intra-operative cholangiogram. We assessed the presentation and management of such patients to refine their management plan.

**Methods:**

Adult patients (>16 years) with an acute gallstone-related disease who had undergone same-stay cholecystectomy from January 2013 to January 2015 were retrospectively assessed. We excluded patients with pre-operative endoscopic CBD exploration.

**Results:**

Among the 612 patients with same-stay cholecystectomy, 399 patients were included in the study, and 213 were excluded because of a pre-operative CBD exploration. Fifty patients (12.5%) presented an image of CBD stone on the intra-operative cholangiogram. Such patients were younger (47 vs. 55 years, *P* = .01) and less likely to present with fever (1 vs. 11.7%, *P* = .04) or signs of cholecystitis on ultrasound (66 vs. 83.7%, *P* = .003). Admission LFTs were higher in patients with an image of a stone. Among the 50 patients with an image on cholangiogram, a stone was confirmed in 26 (52%). Most patients (*n* = 32) underwent post-operative assessment with endoscopic ultrasound (EUS). LFTs did not predict the presence of a confirmed stone. However, the absence of contrast passage into the duodenum was negatively associated with a confirmed stone (*P* = .08), and a filling defect was positively associated with one (*P* = .11). Most confirmed stones were successfully extracted by endoscopic retrograde cholangiopancreatogram (ERCP) (25/26, 96%), except in one patient who needed a per-cutaneous approach because of duodenal diverticuli.

**Conclusions:**

Same-stay cholecystectomy can (and should) be performed even in the presence of moderately abnormal liver function tests. The cholangiogram suspicion of a CBD stone is confirmed in only half of the patients (more often in the presence of a filling defect, and less often with the absence of contrast passage). All stones can be safely treated after surgery (most by ERCP).

**Electronic supplementary material:**

The online version of this article (doi:10.1186/s12893-017-0232-z) contains supplementary material, which is available to authorized users.

## Background

Gallstones are common and affect 10–15% of the adult population. Some 4% of these patients become symptomatic each year, with biliary colics, cholecystitis or cholangitis [1, 2]. In addition, 10–15% of the patients with symptoms also present a common bile duct (CBD) stone [3].

The likelihood of an associated CBD stone and its management have been established by the American Society for Gastrointestinal Endoscopy (ASGE) and Society of American Gastrointestinal and Endoscopic Surgeons (SAGES) guidelines [4]. Globally, patients with normal liver function tests (LFTs) are at low risk (<5%) for a CBD stone and should undergo cholecystectomy first. Those with elevated liver function tests (LFTs) are at increased risk, especially those with total bilirubin >70 μmol/l, which carries a risk for a CBD stone >50%. They should undergo a preliminary exploration of the CBD prior to cholecystectomy. We recently demonstrated that intermediate-risk patients with moderately elevated LFTs (including bilirubin <70 μmol/l) are best treated by upfront same-stay cholecystectomy with intra-operative cholangiogram [5]. This strategy is associated with a decreased length of stay and the need for fewer CBD investigations compared with a primary CBD exploration followed by cholecystectomy. The net effect is that more patients are discovered with an intra-operative CBD stone during same-stay cholecystectomy, and a routine intra-operative cholangiogram is needed [4, 5].

The factors predicting the presence of a CBD stone could still be improved. Such factors could be applied prior to or during surgery. However, a perfect split between patients with and without stone will likely never occur due to the heterogeneity of the signs linked to a CBD stone and because some patients present interval migrations (prior to or during cholecystectomy). Finally, many patients show a spontaneous migration to the duodenum [6]; therefore, a cholangiogram image of a stone does not necessarily translate into a stone on the post-operative assessment.

The aims of our study were therefore to (a) explore factors predicting the presence of a CBD stone image during upfront surgery, to (b) define the predictors of a stone on the post-operative assessment in patients with an image of a CBD stone on cholangiogram, and to (c) explore the efficiency of a post-operative EUS/ERCP management in case of intra-operative CBD stone discovery.

## Methods

### Study design

The study included a retrospective assessment of adult patients (>16 years) with acute gallstone-related disease who had undergone same-stay cholecystectomy from 01.01.2013 to 01.01.2015. Those with a suspected intra-operative CBD stone on cholangiogram were specifically assessed.

Inclusion criteria were: (a) adult patients (>16 years) admitted through the emergency room at the Geneva University Hospitals with a history of acute right upper quadrant pain, (b) radiologically proven gallbladder stone, (c) treatment by cholecystectomy during the same admission, and (d) the absence of CBD investigation prior to cholecystectomy.

Overall, patients presented with (a) prolonged biliary colic, with right upper quadrant pain >6 h, (b) acute cholecystitis with pain, blood signs of inflammation/infection, and a compatible ultrasound assessment, or (c) cholangitis as previously defined [7]. Data were prospectively collected during the hospital stay and analysed at the end of the inclusion period.

### Management

Patients could be categorized into three groups according to their risk of presenting a CBD stone following the ASGE/SAGE guidelines [4]. Low-risk patients demonstrated normal LFTs. High-risk patients included those with serum bilirubin ≥ 70 μmol/l, a visible stone on pre-op imaging (ultrasound (US) or computed tomography (CT)), an ascending cholangitis, and those with bilirubin 30–70 μmol/l and CBD diameter >6 mm. Patients at intermediate risk of a CBD stone included those with abnormal LFTs not fulfilling the aforementioned criteria for high risk of a CBD stone. Based on a recent randomized clinical trial, patients at low and intermediate risk of a CBD stone were managed by cholecystectomy first [5] and represented the target population of patients included in the present study. High-risk patients underwent primary CBD endoscopic exploration followed by cholecystectomy and were not included in the present analysis to improve the population homogeneity.

All patients underwent same-stay laparoscopic cholecystectomy with intra-operative cholangiogram (IOC). Of note, the local policy to systematically performed an IOC is based on safety and didactic reasons, and is aiming at better identifying CBD stones. It is not fully supported by the current literature [8–10]. The cholangiogram looked for a filling defect compatible with a stone, the presence/absence of contrast passage into the duodenum, and a potential iatrogenic bile duct lesion. Patients with a suspected CBD stone on the IOC underwent a post-operative CBD exploration, most often based on endoscopic ultrasound (EUS) followed by endoscopic retrograde cholangiopancreatogram (ERCP) in the case of a confirmed stone. According to the clinical situation, and based on the surgeon’s decision, some patients could be managed with a trans-cystic drain (Escat CH6 drain, Coloplast, Coloplast Group, Denmark).

### Variables of interest

The main outcome of interest was the presence of an image of a CBD stone detected on IOC (filling-defect and/or the absence of contrast passage into the duodenum). We further assessed the patients with a confirmed stone on post-operative EUS.

Studied variables included demographic data (age, gender, body mass index (BMI)), admission data (fever, right upper quadrant pain, signs of cholecystitis on US, admission LFTs), and outcome data (length of hospital stay, conversion rate to a laparotomy, complications according to the Dindo/Clavien classification [11]). In addition, we recorded post-operative LFTs in the patients with an image of a CBD stone on the IOC.

### Statistical analysis and ethics

Demographic and admission data were compared between patients with or without the image of a CBD stone on IOC to look for predictors of intra-operative stone. IOC characteristics and post-operative LFTs were compared between patients with or without a confirmed stone on post-operative EUS to identify predictors of a confirmed CBD stone on post-operative EUS. The groups were compared with Student’s *t*-test and Chi-squared tests. The standard alpha level of .05 indicated statistical significance. Analyses were conducted using SPSS 18.0 (SPSS, Chicago, IL). Ethical approval was obtained from the Institutional Ethical Review Board under the number GE 15–087.

## Results

### Predictors of a CBD stone on IOC

During the study period, 612 adult patients were admitted for an acute gallstone-related disease and underwent a cholecystectomy. Among them, 399 patients were included in the study, and 213 were excluded because they underwent a pre-operative CBD assessment. Most of the patients were female (56.1%), with a mean age of 56 ± 19 years and a mean BMI of 28 ± 6 kg/m^2^ (Table 1). Only two other patients were admitted during the study period, and were not included because they underwent a delayed cholecystectomy in subsequent hospital stay.

**Table 1:** Demographics and presentation comparing patients with or without a common bile duct stone image on cholangiogram

		Total (*N* = 399)	CBD stone (*N* = 50)	No CBD stone (*N* = 349)	*P* value
Gender	Male, No. (%)	175 (43.9%)	16 (32%)	159 (45.6%)	.07
	Female, No. (%)	224 (56.1%)	34 (68%)	190 (54.4%)	
Age (mean ± SD), years		56 ± 19	47 ± 20	55 ± 18	.01
BMI (mean ± SD), kg/m^2^		28 ± 6	29 ± 6	28 ± 5	.58
Clinical presentation	Fever, No. (%)	42 (10.5%)	1 (2%)	41 (11.7%)	.04
	RUQ pain on admission, No. (%)	355 (89%)	42 (84%)	313 (89.7%)	.23
	Associated cholecystitis, No. (%)	325 (81.5%)	33 (66%)	292 (83.7%)	.003
Admission LFTs (mean ± SD)	ASAT, IU/L		167 ± 184	67 ± 146	.001
	ALAT, IU/L		166 ± 197	68 ± 121	.001
	PA, IU/L		123 ± 97	81 ± 48	.005
	GGT, IU/L		245 ± 276	94 ± 139	.001
	Total Bilirubin, μmol/L		26 ± 17	20 ± 12	.01
	Conjugated Bilirubin, μmol/L		18 ± 10	9 ± 7	.002
Abnormal admission LFTs	ASAT (11–42 IU/L), No. (%)	162 (40.6%)	36 (72%)	126 (36.1%)	< .001
	ALAT (9–42 IU/L), No. (%)	163 (40.9%)	34 (68%)	129 (37%)	< .001
	PA (30–125 IU/L), No. (%)	75 (18.8%)	14 (28%)	61 (17.5%)	.07
	GGT (9–35 IU/L), No. (%)	261 (65.4%)	43 (86%)	218 (62.5%)	.001
	Total Bilirubin (7–25 μmol/L), No. (%)	97 (24.3%)	20 (40%)	77 (22.1%)	.006
	Conjugated Bilirubin (2–9 μmol/L), No. (%)	319 (79.9%)	45 (90%)	274 (78.5%)	.06

*BMI* Body Mass Index, *RUQ* Right Upper Quadrant, *CBD* Common Bile Duct, *LFTs* Liver Function Tests, *SD* Standard Deviation, *ASAT* Aspartate Aminotransferase, *ALAT* Alanine Aminotransferase, *PA* Alkaline Phosphatase, *GGT* Gamma-glutamyl Transferase

Among the 399 studied patients with upfront cholecystectomy, 50 (12.5%) presented an image of a CBD stone on IOC (Table 1). Such patients were younger (47 vs. 55 years, *P* = .01) than those without an image of a CBD stone on IOC. In addition, they were less likely to present fever on admission (2 vs. 11.7%, *P* = .04) or an associated cholecystitis on ultrasound (66 vs. 83.7%, *P* = .003). Finally, they demonstrated significantly higher admission LFTs (Table 1). We also assessed similar criteria only looking at patients with intermediate risk of CBD stone (demonstrating similar results as when using the entire cohort) (Additional file 1: Table S1).

In the CBD stone group (*N* = 50), all patients were assessed by IOC. In the No CBD stone group (*N* = 349), the IOC success rate was 81.4% (284/349). In the 65 patients without IOC, the absence of CBD stone was confirmed by MRCP, EUS, ERCP and by following the LFTs until their normalization, and checking the absence of subsequent management for a CBD stone.

Of note the present failure rate is similar to previous ones [9, 10].

### Management of patients with a suspected CBD stone on IOC

**Fig. 1:**
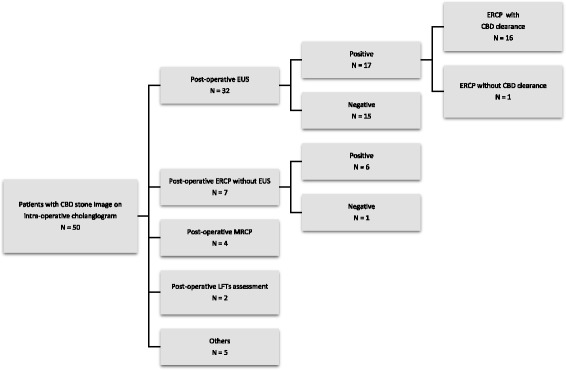
Management of patients with a CBD stone image on intra-operative cholangiogram. *CBD* Common Bile Duct, *LFTs* Liver Function Tests, *EUS* Endoscopic Ultrasound, *ERCP* Endoscopic Retrograde Cholangiopancreatography, *MRCP* Magnetic Resonance Cholangiopancreatography

A total of 50 patients demonstrated an image of a suspected stone on IOC, including 15 (15/50, 30%) with difficult or no contrast passage into the duodenum and 46 (46/50, 92%) with a filling defect compatible with a CBD stone. Among them, 45 patients underwent a post-operative CBD assessment 1.8 ± 1.2 days after surgery (Fig. 1). Among them, 32 underwent post-operative EUS, which confirmed the presence of a CBD stone in 17 patients (53.1%). All but one obtained a successful CBD clearance by ERCP. The only failure was linked to the presence of a duodenal diverticuli, and the stone was extracted by interventional radiology. Seven patients underwent post-operative ERCP without previous EUS. Six of them had a confirmed CBD stone, all successfully extracted. In addition, four patients underwent post-operative magnetic resonance cholangiopancreatography (MRCP), all negative. Two patients were followed with normalized LFT levels, as they refused any further investigation.

One patient underwent intra-operative ERCP with successful stone extraction. Two underwent laparoscopic stone extraction utilizing a Dormia basket. Two patients had a cholangiography through a transcystic drain left during surgery before EUS/ERCP. As this examination was negative, no further endoscopic assessment was performed, and the patients had drain removal a few weeks later.

Linked to the need for added investigation/intervention, patients with an image of a CBD stone on cholangiogram showed a longer length of stay, but the rates of major (stage 3 and 4) complications were not significantly different (6/50, 12% vs. 22/349, 6%, *P* = .14, (Table 2)).

**Table 2:** Outcomes

		Total	CBD stone	No CBD stone	*P* value
		(*N* = 399)	(*N* = 50)	(*N* = 349)	
Conversion from LS to LT, No. (%)		8 (2%)	1 (2%)	7 (2%)	.998
Laparotomy first, No. (%)		3 (0.8%)	1 (2%)	2 (0.6%)	.275
LOS (mean ± SD), days		6.9 ± 4	8.8 ± 4.7	6.6 ± 3.8	.003
Complications (Dindo-Clavien classification)	Grade III, No	21	4	17	.35
	Grade IV, No	7	2	5	.2

*LOS* Length of Stay, *SD* Standard Deviation, *LS* Laparoscopy, *LT* Laparotomy

In the CBD stone group, one patient (1/50, 2%) has been converted from laparoscopy to laparotomy because of adhesions and a stomach sero-muscular lesion. In addition, another patient underwent a laparotomy first due to a patent foramen ovale. In the No CBD stone group, 7 patients had a conversion (7/349, 2%), due to adhesions, severe pediculitis, haemorrhage, small bowel perforation, lack of identification of the cystic duct and necrotized gallbladder with peritonitis. In addition, two other patients underwent a laparotomy first due to severe heart failure and septic shock.

### Predictors of a CBD stone on post-operative CBD assessment

To identify predictors of confirmed CBD stones on post-operative EUS (only this group was used for a better homogeneity), the 15 patients with no sign of a remaining stone were compared with the 17 with a confirmed stone. The time between surgery and EUS was similar between the two groups (Table 3). LFTs assessed prior to EUS did not predict the presence of a stone on EUS (Table 3). An image of a filling defect on IOC compatible with a CBD stone tended to predict the presence of a confirmed stone (*P* = .11). Conversely, the absence of contrast passage into the duodenum tended to be associated with an absence of a stone on EUS (*P* = .08).

**Table 3:** Comparison of patients with positive versus negative post-operative endoscopic ultrasound

		Positive EUS	Negative EUS	*P* value
		(*N* = 17)	(*N* = 15)	
LFTs before EUS	ASAT, IU/L	119 ± 120	100 ± 87	.62
	ALAT, IU/L	151 ± 194	167 ± 117	.79
	PA, IU/L	128 ± 89	86 ± 43	.18
	GGT, IU/L	290 ± 265	260 ± 233	.76
	Total Bilirubin, μmol/L	24 ± 15	33 ± 28	.28
	Conjugated Bilirubin, μmol/L	21 ± 12	31 ± 21	.34
Pancreatic tests before EUS	Lipase, IU/L	26 ± 9	108 ± 192	.3
Intra-operative cholangiogram	Filling defect present, No.	16	11	.11
	absent, No.	1	4	
	Contrast in duodenum present, No.	14	8	.08
	absent, No.	3	7	
Delay between surgery and post-operative EUS, days		1.8 ± 0.8	1.9 ± 1.4	.81

*LFTs* Liver Function Tests, *EUS* Endoscopic Ultrasound *ASAT* Aspartate Aminotransferase, *ALAT* Alanine Aminotransferase, *PA* Alkaline Phosphatase, *GGT* Gamma-glutamyl Transferase

### Impact of a trans-cystic drain

Of the patients described earlier, five (5/50, 10%) were managed with a transcystic drain. Among them, 3 (3/5, 60%) had no contrast passage into the duodenum. The delay between surgery and post-operative CBD investigation was significantly shorter for the group of patients with a transcystic drain than for those without a drain (1 ± 0.4 vs. 2 ± 1.3 days, *P* = .003). There was no significant difference in the length of stay (LOS) (11 ± 8 vs. 9 ± 4 days, *P* = .56). Complication rates were similar, but one patient developed a stage 4 Dindo/Clavien complication due the displacement of the trans-cystic drain (Table 4).

**Table 4:** Usefulness and safety of transcystic drainage in patients with an intra-operative suspicion of common bile duct stone

		Patients with transcystic drainage (*N* = 5)	Patients without transcystic drainage (*N* = 45)	*P* value
Duodenal passage of contrast product during IOC	Difficult duodenal passage, No.	2 (40%)	4 (9%)	.04
	No duodenal passage, No.	3 (60%)	4 (9%)	.002
Delay between surgery and post-operative CBD assessment, days		1 ± 0.4	2 ± 1.3	.003
Length of stay (mean ± SD), days		11 ± 8	9 ± 4	.56
Length of transcystic drainage (mean ± SD), days		39 ± 22	0	
Complications (Dindo-Clavien classification)	Grade I, No.	0	7	.34
	Grade II, No.	0	3	.55
	Grade III, No.	0	4	.49
	Grade IV, No.	1	1	.05

*IOC* Intra-operative Cholangiogram, *CBD* Common Bile Duct, *SD* Standard Deviation

## Discussion

The risk of a CBD stone is classically defined by a combination of clinical, biological and radiological parameters [4]. The present study assessed patients without previous CBD exploration and confirmed the value of LFTs, and clinical variables such as fever, as well as signs of cholecystitis on US [12]. In addition, women were more likely to present a stone.

Despite these known risk factors, our local policy is to perform an IOC in all patients undergoing cholecystectomy because models predicting the presence of a stone are not accurate enough [10–12]. Alternatively, IOC appears as efficient as ERCP in predicting the presence of a stone [13, 14]. Additionally, it bears a didactic value, training younger surgeons at approaching the CBD for a potential exploration. Finally, IOC also helps to detect an iatrogenic bile duct lesion [15]. We acknowledge, however, that patients with normal LFTs undergoing elective cholecystectomy do not necessarily need an IOC. Conversely, we strongly recommend following the IOC path in patients undergoing cholecystectomy for acute gallstone-related diseases, especially in the case of abnormal LFTs.

Overall, an intra-operative stone was suspected in 12.5% (50/399) of the studied patients, based on IOC abnormalities. The preliminary injection of a limited quantity of contrast agent helps to detect a filling defect, while a stone can be less visible after the injection of a large volume of contrast agent. Of note, manoeuvres such as changing the table position and/or injecting saline help in the differential diagnosis with an air bubble. The second type of abnormality is the lack or a difficult contrast passage into the duodenum, which may be due to a trapped CBD stone, an Oddi’s sphincter spasm, or a lesion of the sphincter (neoplasia/inflammation).

Only half of the suspected stones could be confirmed post-operatively. Based on the current data, it remains difficult to predict which patients will finally present a confirmed stone. Post-operative LFTs have no predictive power. LFTs may remain increased because of the previous injection of contrast during IOC or because a liver lesion occurred during the removal of the gallbladder from its bed. Only the presence of a filling defect tends to be predictive of a remaining CBD stone, whereas the absence of contrast passage into the duodenum is not. Most contrast passage alterations are likely linked to spasms. The net results of these observations are that a post-operative CBD assessment is required in all patients with an IOC abnormality and that LFTs do not necessarily need to be repeated after surgery due to their low predictive value.

We favour the use of post-operative EUS (+/-ERCP) in patients with an image of a stone during same-stay cholecystectomy. In fact, EUS accuracy is very high (near 97%) as are the sensitivity (71–100%), specificity (67–100%) and the positive or negative predictive values [16–19].

Such a strategy allowed the extraction of all stones by ERCP (with the exception of one patient treated by interventional radiology). As an alternative, laparoscopic CBD exploration could also be used. However, it can require the use of laparoscopic choledochotomy, with a risk of bile leak up to 15%, especially in patients with non-dilated CBD, and in the presence of inflammation [20]. In addition, the ERCP stone clearance rate appears higher than that after laparoscopic bile duct exploration [21, 22].

Another option would have been to perform ERCP during surgery (with or without rendezvous) [23]. Here again, we have not selected this strategy primarily because half of the patients do not show a confirmed stone on post-operative tests (and would have undergone unnecessary ERCP). Secondarily, emergency intra-operative ERCP is difficult to organize because of endoscopist availability and the positioning of the patient being non-standard for ERCP. The present study is limited by its retrospective nature and its potential for type 2 errors. However, it provides a real-life assessment of the proposed management strategy of patients at risk of CBD stones.

The present study is limited by its retrospective nature and its potential for type 2 errors. However, it provides a real-life assessment of the proposed management strategy of patients at risk of CBD stones.

## Conclusion

As a whole, the present study confirms that same-stay cholecystectomy can (and should) be performed even in the presence of moderately abnormal liver function tests. The cholangiogram suspicion of a CBD stone can be confirmed in only half of the patients (more often in the presence of a filling defect, and less often with the absence of contrast passage). All stones can be safely treated after surgery (most by ERCP).

## Additional file

Additional file 1: Table S1. Demographics and presentation comparing intermediate risk patients with or without a common bile duct stone image on cholangiogram. Assessment of demographic and clinical criteria considering only the patients at intermediate risk of CBD stone. The results were similar to those using the entire cohort. (PDF 15 kb)

## Abbreviations

ALAT: Alanine aminotransferase
ASAT: Aspartate aminotransferase
ASGE: American Society for Gastrointestinal Endoscopy
BMI: Body mass index
CBD: Common bile duct
CT: Computed tomography
ERCP: Endoscopic retrograde cholangiopancreatogram
EUS: Endoscopic ultrasound
GGT: Gamma-glutamyl transferase
IOC: Intra-operative cholangiogram
LFTs: Liver function tests
LOS: Length of stay
LS: Laparoscopy
LT: Laparotomy
MRCP: Magnetic resonance cholangiopancreatography
PA: Alkaline phosphatase
RUQ: Right upper quadrant
SAGES: Society of American Gastrointestinal and Endoscopic Surgeons
SD: Standard deviation
US: Ultrasound
