# Supplementary material for: Management of suspected common bile duct stones on cholangiogram during same-stay cholecystectomy for acute gallstone-related disease
Source: BMC Surg. 2017 Apr 17;17:39. doi: 10.1186/s12893-017-0232-z (PMC5392997; doi:10.1186/s12893-017-0232-z)
Supplement: Additional file 1: Table S1. — Demographics and presentation comparing intermediate risk patients with or without a common bile duct stone image on cholangiogram. Assessment of demographic and clinical criteria considering only the patients at intermediate risk of CBD stone. The results were similar to those using the entire cohort. (PDF 15 kb) [file 12893_2017_232_MOESM1_ESM.pdf]

Supplementary Table 1: Demographics and presentation comparing intermediate risk patients with or without a common bile duct stone image on cholangiogram					
		Total (N=399)	CBD stone (N=50)	No CBD stone (N=349)	P value
Intermediate risk		N=126	N=29	N=97	
Gender	Male, No. (%)	51 (40.5%)	8 (27.6%)	43 (44.3%)	.107
	Female, No. (%)	75 (59.5%)	21 (72.4%)	54 (55.7%)	
Age (mean ± SD), years		53 ± 21	46 ± 24	55 ± 20	.058
BMI (mean ± SD), kg/m^2		28 ± 5	30 ± 6	27 ± 5	.05
Clinical presentation	Fever, No. (%)	13 (10.3%)	1 (3.4%)	12 (12.4%)	.166
	RUQ pain on admission, No. (%)	108 (85.7%)	23 (79.3%)	85 (87.6%)	.261
	Associated cholecystitis, No. (%)	86 (68.3%)	16 (55.2%)	70 (72.2%)	.085
Admission LFTs (mean ± SD)	ASAT, IU/L		219 ± 194	87 ± 116	.002
	ALAT, IU/L		243 ± 222	92 ± 139	.002
	PA, IU/L		162 ± 113	90 ± 52	.003
	GGT, IU/L		332 ± 308	122 ± 181	.002
	Total Bilirubin, µmol/L		32 ± 19	23 ± 14	.017
	Conjugated Bilirubin, µmol/L		20 ± 10	11 ± 8	.006
Abnormal admission LFTs	ASAT (11-42 IU/L), No. (%)	49 (38.9%)	3 (10.3%)	46 (47.4%)	< .001
	ALAT (9-42 IU/L), No. (%)	52 (41.3%)	3 (10.3%)	49 (50.5%)	< .001
	PA (30-125 IU/L), No. (%)	91 (72.2%)	15 (51.7%)	76 (78.4%)	.005
	GGT (9-35 IU/L), No. (%)	35 (27.8%)	1 (3.4%)	34 (35.1%)	< .001
	Total Bilirubin (7-25 µmol/L), No. (%)	75 (59.5%)	13 (44.8%)	62 (63.9%)	.066
	Conjugated Bilirubin (2-9 µmol/L), No. (%)	27 (21.4%)	3 (10.3%)	24 (24.7%)	.097

BMI = Body Mass Index, RUQ = Right Upper Quadrant, CBD = Common Bile Duct, LFTs = Liver Function Tests, SD = Standard Deviation.

ASAT = Aspartate Aminotransferase, ALAT = Alanine Aminotransferase, PA = Alkaline Phosphatase, GGT = Gamma-glutamyl Transferase.
